# Supplementary material for: The causal relationship between the human gut microbiota and pyogenic arthritis: a Mendelian randomization study
Source: Front Cell Infect Microbiol. 2024 Nov 26;14:1452480. doi: 10.3389/fcimb.2024.1452480 (PMC11629706; doi:10.3389/fcimb.2024.1452480)
Supplement: Supplementary file 1 [file DataSheet1.zip › mendelian test/ebi-a-GCST90027733.csv_leave_one_out.pdf]

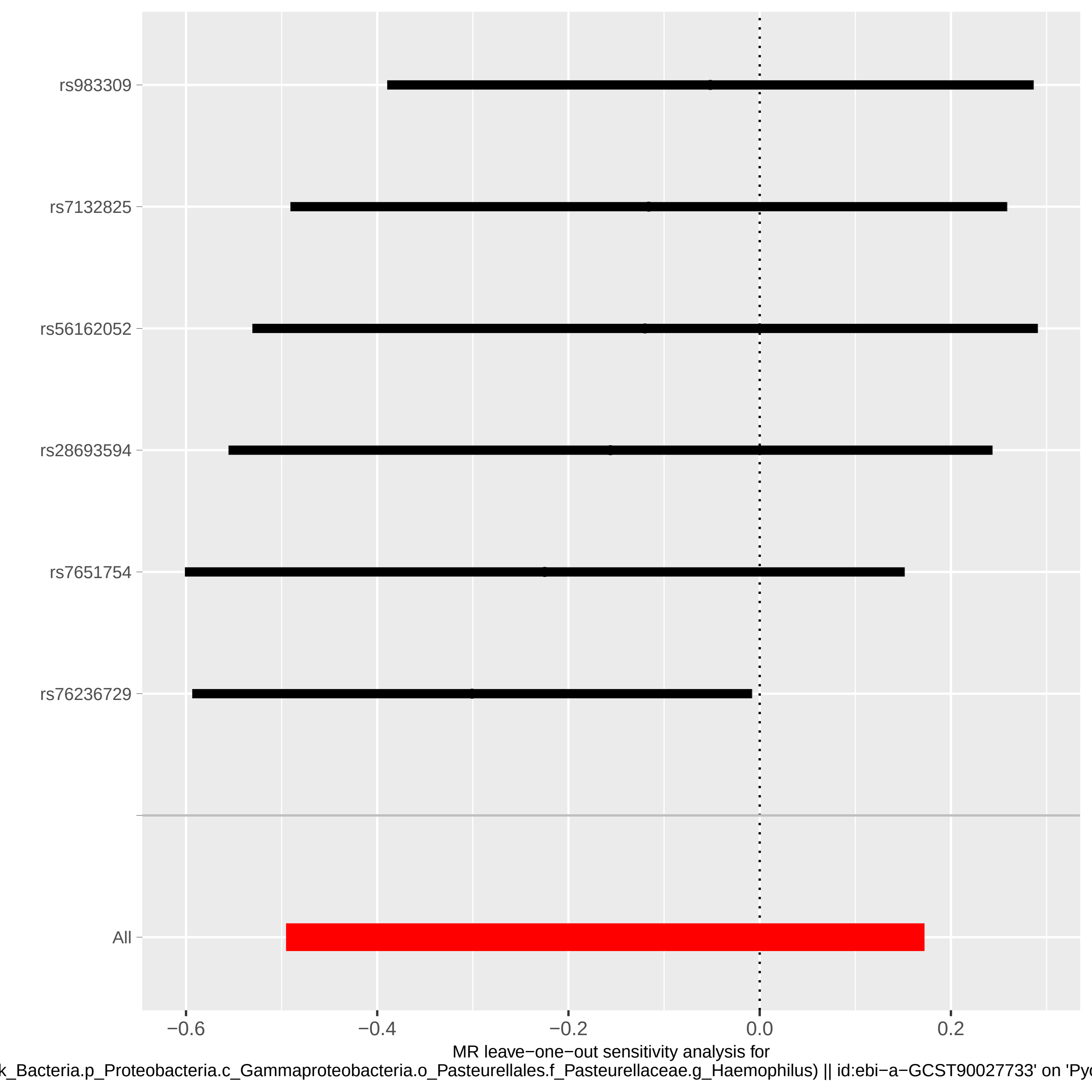
rs983309
rs7132825
rs56162052
rs28693594
rs7651754
rs76236729
All
−0.6
−0.4
−0.2
0.0
0.2
MR leave−one−out sensitivity analysis for
k_Bacteria.p_Proteobacteria.c_Gammaproteobacteria.o_Pasteurellales.f_Pasteurellaceae.g_Haemophilus) || id:ebi−a−GCST90027733' on 'Py
